# FUNCTION FOCUSED CARE FOR ACUTE CARE: OVERCOMING RECRUITMENT, MEASUREMENT, AND IMPLEMENTATION POST-COVID-19

**DOI:** 10.1093/geroni/igac059.1532

**Published:** 2022-12-20

**Authors:** Barbara Resnick

## Abstract

To help patients spend more time engaged in physical activities and avoid the complications that occur Function Focused Care for Acute Care was developed (FFC-AC-EIT). FFC-AC-EIT includes the implementation of four steps: (1) Environment and policy assessments; (2) Education of staff; (3) Establishing patient goals; and (4) Mentoring and motivating of staff, patients, and families. A total of 600 patients from 12 hospitals will be included. Eligibility of patients is based on being 55 years of age and older, admitted for a medical reasons excluding COVID-19, and demonstrating evidence of dementia. Outcome measures are obtained at baseline, discharge, 1, 6 and 12 months post discharge and include physical function, physical activity, pain and pain management, psychological and behavioral symptoms associated with dementia, delirium and adverse events (falls, rehospitalizations, nursing home admissions). Due to COVID-19 innovative approaches were implemented to be able to initiate and continue with the study. These included: identifying potential participants that were COVID-19 free off site; transitioning some intervention activities with staff to online; completing verbal consent with proxies versus face to face; adjusting follow up MotionWatch 8 deliveries and placements to be done without face to face interaction; and adjusting recruitment time periods and intervention activities to fit with intermittently high periods of COVID-19. This symposium will describe intervention challenges, solutions and lessons learned, describe an effective process and measurement model for identification of participants with dementia; and provide optimal ways to measure pain and physical activity among older adults with dementia.

